# Polymyositis Presenting as Rhabdomyolysis After the Initiation of Omeprazole

**DOI:** 10.7759/cureus.8125

**Published:** 2020-05-14

**Authors:** Jonathan K Jakubowski, Rosemina Patel, Venkata Buddharaju

**Affiliations:** 1 Internal Medicine, Chicago Medical School - Rosalind Franklin University of Medicine and Science, North Chicago, USA; 2 Nephrology, Chicago Medical School - Rosalind Franklin University of Medicine and Science, North Chicago, USA

**Keywords:** rhabdomyolysis, polymyositis, proton pump inhibitor

## Abstract

Rhabdomyolysis is a clinical syndrome with a wide range of presentations; it results in muscle necrosis and release of intracellular muscle contents into the circulation. Inflammatory myopathies are a rare cause of rhabdomyolysis. We present a case of a 46-year-old male with a two-week history of progressively worsening diffuse muscle pain after he had been prescribed omeprazole one month prior. A creatine phosphokinase (CPK) elevation was noted, which persisted despite treatment with IV fluids, sodium bicarbonate, and close correction of electrolytes. Further workup, including autoimmune and infectious etiologies, was notable for elevated antinuclear antibodies (ANA), erythrocyte sedimentation rate (ESR), and C-reactive protein (CRP). Furthermore, a muscle biopsy showed evidence of endomysial inflammatory cells, consistent with a diagnosis of polymyositis. Steroids were initiated with significant improvement in symptoms and a decrease in CPK levels. The patient was discharged on a tapering dose of steroids and, on follow-up with the rheumatologist, transitioned to methotrexate with control of symptoms. In patients with rhabdomyolysis who do not respond to first-line therapy, obtaining a detailed medication history and screening with ANA and ESR are encouraged. Given the link between medication and autoimmune disease, clinicians should consider autoimmune myopathy in the differential for cases with persistently elevated creatine kinase. Prompt diagnosis with early initiation of immunosuppressive medication may improve outcomes and avoid complications associated with untreated rhabdomyolysis or polymyositis.

## Introduction

Rhabdomyolysis is a clinical syndrome that results in muscle necrosis and the release of muscle cell contents into the circulation, most notably myoglobin. Rhabdomyolysis is associated with a wide-spectrum manifestation, from remaining clinically silent as a benign course to a severe systemic presentation causing pigment-induced nephropathy [1]. It may arise from a traumatic or non-traumatic etiology including toxins, electrolyte disturbances, infection, medications, immobilization, seizures, and, rarely, autoimmune myopathies. Medications such as statins have been documented to contribute to the development of autoimmune myopathies [2-4]. However, only a few cases of proton pump inhibitor (PPI)-induced myopathies have been reported. Inflammatory myopathies are a rare cause of rhabdomyolysis. We present a unique case of a patient who initially presented with rhabdomyolysis, later with hemoptysis, and was eventually diagnosed with polymyositis.

## Case presentation

A 46-year-old Hispanic male presented in late summer with three days of abdominal pain and diarrhea. He also endorsed a two-week history of progressively worsening diffuse muscle pain, notably worse in the lower extremities. He denied any trauma, recent illness, or any relevant family medical history. His medical history included gastroesophageal reflux disease diagnosed one month ago, for which omeprazole had been prescribed, which had led to an improvement of his heartburn. On examination, vital signs were within normal limits and he had mild tenderness to palpation of the abdomen. Extremities showed decreased muscle strength, which was more profound in the lower extremities; however, he remained neurologically intact. Initial labs showed aspartate aminotransferase (AST) of 494, alanine aminotransferase (ALT) 290, troponin I of 0.36, creatine kinase-MB (CKMB) 915.5 with a relative index of 11.5, and a creatine phosphokinase (CPK) of 7974. Urine dipstick was positive for blood; however, no RBCs were seen on microscopy. A urine drug screen was negative. His electrocardiogram showed normal sinus rhythm with no ST-T wave changes. A CT of the abdomen was obtained, which was unremarkable.

The patient was admitted and started on aggressive IV fluids for rhabdomyolysis and non-ST elevated myocardial infarction (NSTEMI). His home medication was held on admission. To rule out acute coronary syndrome, the patient underwent a cardiac workup with an echocardiogram, which showed a normal ejection fraction and no wall motion abnormalities; he also underwent a nuclear stress test later, which was negative for myocardial ischemia. Elevated troponin was therefore suggested to be related to rhabdomyolysis. The patient was still symptomatic with myalgia and CPK remained elevated above 6000 despite adequate hydration and addition of a bicarbonate infusion. On hospital day six, the patient underwent further evaluation for the persistent elevation of CPK. Infectious workup including hepatitis A, B, and C returned negative. ANA was noted to be greater than 1:640 with a speckled pattern; CRP of 2.83 and ESR of 44 were also observed. An autoimmune cause for rhabdomyolysis was suspected. A trial of steroids with methylprednisolone 40 mg IV was given, with remarkable improvement of symptoms. The patient’s CPK declined to 4000, and he was discharged on a tapering dose of prednisone for suspected autoimmune myositis. The patient returned less than 24 hours later with a similar presentation with a new onset of hemoptysis.

**Figure 1:**
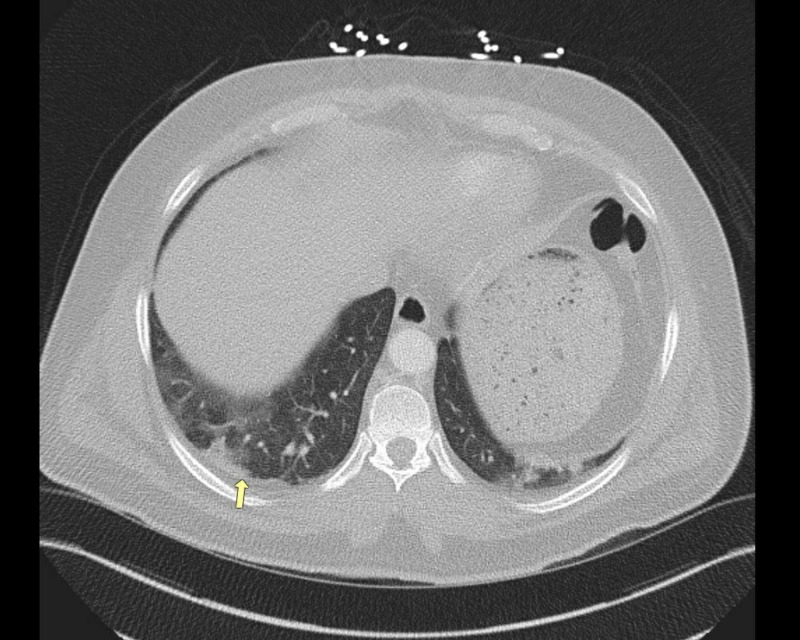
CT angiogram of the chest, with nodular opacities (arrow) CT: computed tomography

**Figure 2:**
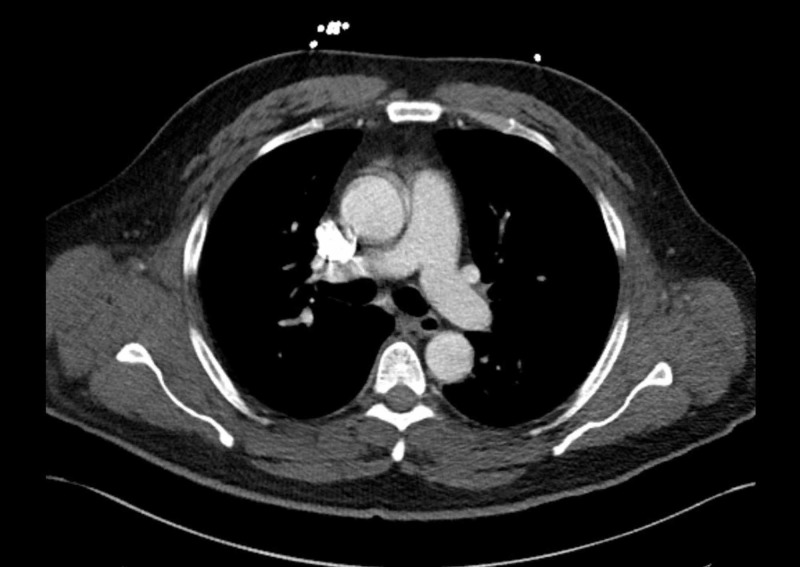
CT angiogram of the chest The image shows no evidence of pulmonary embolism from the main pulmonary artery through the bifurcation to the right and left pulmonary arteries CT: computed tomography

**Figure 3:**
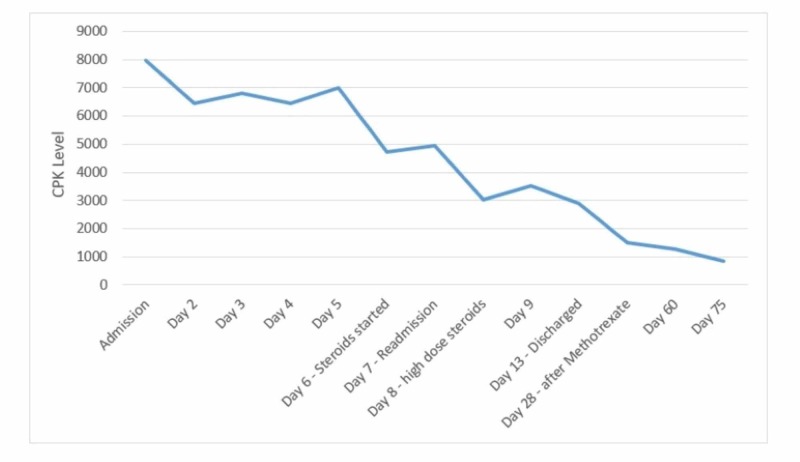
Muscle enzyme trends - from diagnosis to resolution CPK: creatine phosphokinase

During the second admission, he was given 1 mg/kg of IV methylprednisolone. Omeprazole was again held on admission with a transition to famotidine. Repeat laboratory data showed a CPK of 5026, serum aldolase of 81.3, and urine dipstick positive for blood; however, no red blood cells on microscopy were observed. CT angiogram of the chest was obtained, which was negative for pulmonary embolism but showed bilateral parenchymal nodular opacities (Figure 1, 2). The patient underwent bronchoscopy, which showed normal endobronchial anatomy without gross hemorrhage or hemoptysis, and with cytopathology showing hemosiderin-laden macrophages, consistent with diffuse alveolar hemorrhage (DAH). A complete serologic panel was obtained, which came back negative (Table 1). For a definitive diagnosis of inflammatory myositis, the patient underwent a muscle and skin biopsy of the right quadriceps. The skin biopsy showed no significant histological abnormalities; however, the muscle biopsy showed evidence of endomysial inflammatory cells in the muscle fascicles with regeneration, consistent with a diagnosis of polymyositis. There was no evidence of necrosis, rimmed vacuoles, or perivascular inflammatory changes. High- dose steroids were continued with a resolution of hemoptysis and muscle pain. CPK continued to trend down with no further evidence of myoglobinuria (Figure 3). Omeprazole was discontinued indefinitely. He was discharged on a tapering dose of oral prednisone.

**Table 1:** Autoimmune workup of the patient ANA: antinuclear antibody; Anti-U1 RNP: anti-U1 ribonucleoprotein; Anti-SSA 52 (Ro): anti-Sjogren's syndrome-related antigen A/anti-Ro; Anti-U3 RNP: anti-U3 ribonucleoprotein; Anti-GBM: anti-glomerular basement membrane; Anti-SCL-70: anti-topoisomerase I; Anti-dsDNA: anti-double-stranded DNA; ANCA: anti-neutrophil cytoplasmic antibody; IFA: immunofluorescence assay

Antibody	Result	Reference range
ANA titer	>1:640	
ANA pattern	Speckled	
Anti-U1 RNP	5	0-40 AU/mL
Anti-SSA 52 (Ro)	9	0-40 AU/mL
Anti-Jo 1	3	0-40 AU/mL
Anti-U3 RNP	Negative	
Anti-GBM	0	0-19 AU/mL
Anti-SCL-70	8	0-40 AU/mL
Anti-dsDNA	Negative	
ANCA IFA	<1:20	

**Figure 4:**
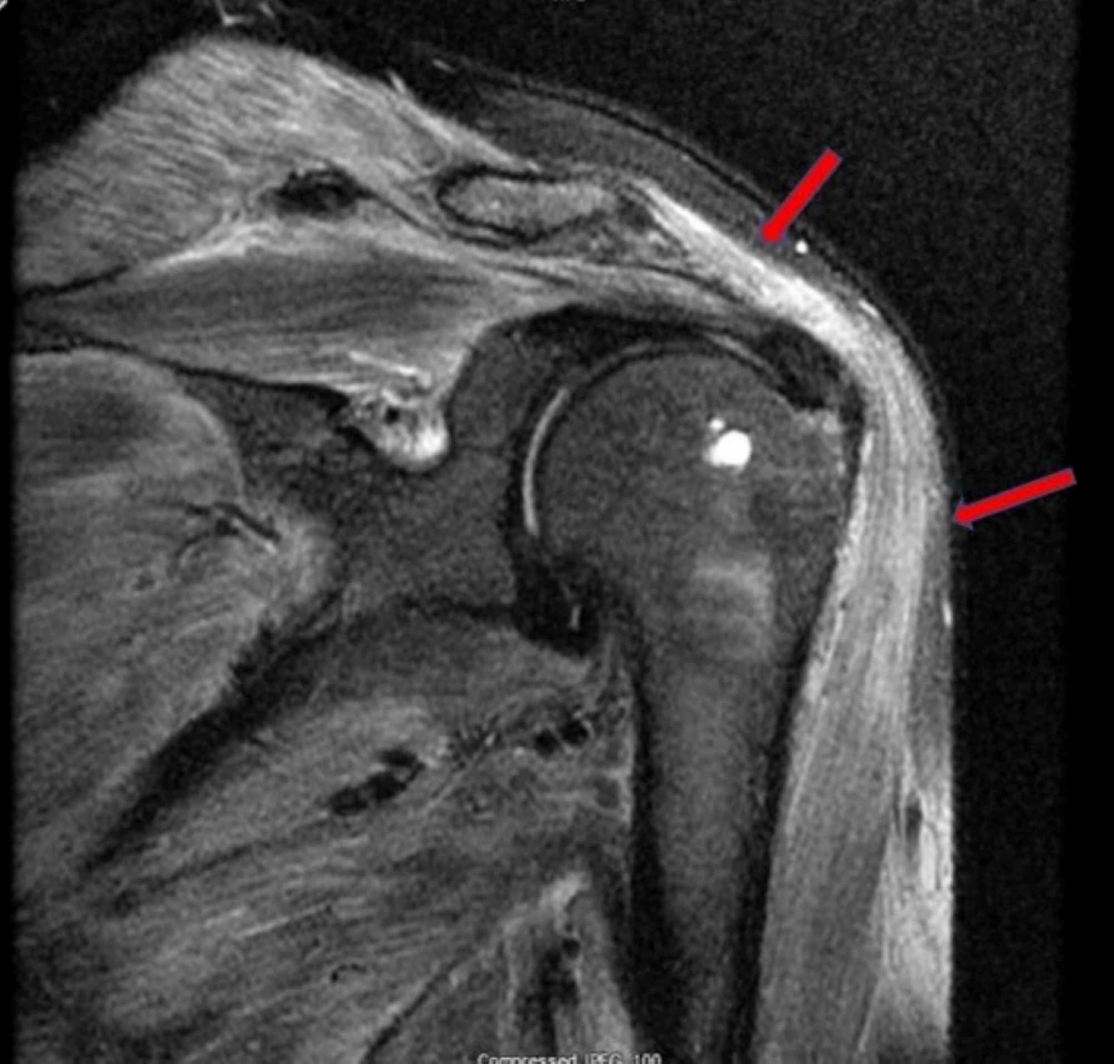
MRI of the left shoulder without contrast The image shows diffuse muscle edema on T2-weighted image (arrows) MRI: magnetic resonance imaging

After discharge, the patient had an MRI of the left shoulder without contrast, which demonstrated a diffuse increase in T2 signal of the cuff muscles, suggestive of edema, and possibly active inflammation (Figure 4). He was seen by a rheumatologist who started him on methotrexate. The patient’s symptoms remained well controlled after dose titration. A repeat CPK two months later was noted to be 1271 with other muscle enzymes also showing a downward trend.

## Discussion

Polymyositis is a subtype of idiopathic inflammatory myopathy (IIM). The main symptom of the condition is symmetric proximal muscle weakness developing over three to six months. Other findings include elevation in muscle enzymes, muscle-specific antibodies, muscle biopsy showing endomysial inflammation with infiltrate rich in CD8 T cells, and increased major histocompatibility complex class I (MHC-1) expression. Diagnostic methods that aid in the diagnosis of polymyositis consist of electromyography indicating myopathy and MRI with T2-weighted images with fat suppression to identify edema. Our patient met three out of the four criteria in the Bohan and Peter classification for polymyositis, indicating probable polymyositis [5]. According to the European League Against Rheumatism and the American College of Rheumatology EULAR/ACR classification criteria for IIM, our patient had a score of 6.5, which is below the cut-off for the diagnosis of IIM; however, it should be kept in mind that the score does not include MRI and electromyography (EMG) data [6]. Our patient had MRI evidence of proximal muscle inflammation, which would have increased the probability of IIM.

Polymyositis is a rare cause of rhabdomyolysis as it typically presents insidiously. One retrospective study by Melli et al. reviewed 475 patients with rhabdomyolysis, of which only 27 patients were identified to have IIM (21 patients with polymyositis, four with dermatomyositis, and two with overlap syndrome) [7]. Individual case reports have been published in literature where IIM presented with rhabdomyolysis, as seen with our patient [8-10].

There is also evidence of PPIs increasing the risk of rhabdomyolysis in a few case series and reports. A review of individual case reports by Duncan and Howden identified seven cases of rhabdomyolysis associated with PPI. Two of these seven patients were taking HMG-CoA reductase inhibitors, which are known to cause varying degrees of myopathy. None of the patients were re-challenged with PPIs [11]. Hence, there is some evidence linking PPIs to rhabdomyolysis, although a causal relationship cannot be established. Another review study published by Clark et al. analyzed 35 cases of rhabdomyolysis associated with PPI treatment; 12 of these patients were concomitantly taking HMG-CoA reductase inhibitors. PPIs were re-introduced in only one patient in whom rhabdomyolysis did not re-occur. In the same study, there were 27 case reports in which PPI was associated with an IIM [12]. Of those cases, six were confirmed polymyositis, five were related to omeprazole, with one due to pantoprazole. In about 45% of the reports, where time to onset was given, the symptoms developed during the first three months of treatment.

Several mechanisms have been proposed for PPIs as a cause of rhabdomyolysis. PPIs are metabolized through the hepatic cytochrome P450 enzymes, with CYP2C19 having the primary role with inhibition of CYP3A4 to some extent. Many other drugs, like statins, undergo metabolism by CYP3A4. These pharmacokinetic interactions can increase exposure to statins, thus increasing the risk of rhabdomyolysis. CYP2C19 polymorphisms contribute to the reduced metabolism of PPIs [13]. A study by Goktas et al. showed that the CYP2C19 activity of the patients with Behçet’s disease was significantly lower as compared to healthy subjects [14]. PPIs act by binding to H+/K+-ATPase in gastric parietal cells, and theoretically may also bind to other cells like vascular smooth muscle cells, which can alter the intracellular environment and predispose cells to degrade. Another proposed mechanism may be related to decreased metabolism of reactive oxygen species (ROS). A study by May et al. showed reduced cytochrome P‐450 activity causing a decline in ROS metabolism, which may play a significant role in scleroderma pathogenesis [15]. Previous studies had found that pathogenesis of autoimmune diseases might be related to increased ROS [15]. Another study by Sivakumar and Dalakas provided evidence that autoimmune disease due to omeprazole use may be caused by an alteration in the catabolism of nuclear chromatin by omeprazole, similar to what has been proposed as a cause of drug-induced lupus [16].

Given our patient’s presentation after initiating omeprazole, we now believe he may have had a CYP2C19 polymorphism, which triggered the development of polymyositis, although we were unable to prove it at the time. Further data are needed to confirm our suspicion. Given the link between PPIs and autoimmune disease, we suggest keeping a high index of suspicion for inflammatory myopathies by obtaining a detailed medication history and screening with an ANA and inflammatory markers in patients with rhabdomyolysis who do not respond to first-line therapy.

It is unclear why our patient developed alveolar hemorrhage. DAH in autoimmune disorders is usually seen in systemic vasculitis syndromes, mixed connective tissue disorders (MCTD), and anti-glomerular basement membrane antibodies. Our patient did not show any serological evidence of other systemic diseases, making an overlap syndrome less likely. While lung is the most common extra-muscular site of involvement in inflammatory myopathies, pulmonary manifestations usually include interstitial lung disease. Reports of DAH in IIMs, although rare, are found in the literature [17,18]. Most cases of DAH have been associated with increased mortality due to respiratory failure unless promptly treated with immunosuppressive therapy [19]. In patients who underwent an autopsy, biopsy showed pulmonary capillaritis [20]. Therefore, prompt treatment with methylprednisone and/or IV cyclophosphamide should be started in patients with inflammatory myopathies in whom DAH is present. Fortunately, our patient did not develop overt respiratory failure, probably due to being on prednisone therapy on the second presentation, and did not show any evidence of DAH on follow-up.

## Conclusions

Clinicians should consider an autoimmune myopathy in the differential for patients with persistently elevated CPK who are on medication linked to the diagnosis. A prompt diagnosis followed by early initiation of immunosuppressive medication may improve outcomes and avoid complications associated with untreated rhabdomyolysis or polymyositis. Given that PPIs are easily accessible over the counter and are among the most frequently prescribed medications, more studies are needed to explore their relationship with autoimmune myopathies.
